# Transmission of Infectious *Vibrio cholerae* through Drinking Water among the Household Contacts of Cholera Patients (CHoBI7 Trial)

**DOI:** 10.3389/fmicb.2016.01635

**Published:** 2016-10-18

**Authors:** Raisa Rafique, Mahamud-ur Rashid, Shirajum Monira, Zillur Rahman, Md. Toslim Mahmud, Munshi Mustafiz, K. M. Saif-Ur-Rahman, Fatema-Tuz Johura, Saiful Islam, Tahmina Parvin, Md. Sazzadul I. Bhuyian, Mohsena B. Sharif, Sabita R. Rahman, David A. Sack, R. Bradley Sack, Christine M. George, Munirul Alam

**Affiliations:** ^1^International Center for Diarrhoeal Disease ResearchBangladesh, Dhaka, Bangladesh; ^2^Department of Microbiology, Dhaka UniversityDhaka, Bangladesh; ^3^Department of International Health, Johns Hopkins Bloomberg School of Public HealthBaltimore, MD, USA

**Keywords:** cholera, *Vibrio cholerae*, clonal transmission, household contact, PFGE, Bangladesh

## Abstract

Recurrent cholera causes significant morbidity and mortality among the growing population of Dhaka, the capital city of Bangladesh. Previous studies have demonstrated that household contacts of cholera patients are at >100 times higher risk of cholera during the week after the presentation of the index patient. Our prospective study investigated the mode of transmission of *Vibrio cholerae*, the cause of cholera, in the households of cholera patients in Dhaka city. Out of the total 420 rectal swab samples analyzed from 84 household contacts and 330 water samples collected from 33 households, *V. cholerae* was isolated from 20%(17/84) of household contacts, 18%(6/33) of stored drinking water, and 27%(9/33) of source water samples. Phenotypic and molecular analyses results confirmed the *V. cholerae* isolates to be toxigenic and belonging to serogroup O1 biotype El Tor (ET) possessing cholera toxin of classical biotype (altered ET). Phylogenetic analysis by pulsed-field gel electrophoresis (PFGE) showed the *V. cholerae* isolates to be clonally linked, as >95% similarity was confirmed by sub-clustering patterns in the PFGE (*Not*I)-based dendrogram. Mapping results showed cholera patients to be widely distributed across 25 police stations. The data suggesting the transmission of infectious *V. cholerae* within the household contacts of cholera patients through drinking water underscores the need for safe water to prevent spread of cholera and related deaths in Dhaka city.

## Introduction

Severe cholera without adequate rehydration can have a case fatality rate of up to 50% (Sack et al., 2004). Worldwide an estimated 3–5 million cholera cases occur annually (WHO, 2015). *Vibrio cholerae* is the etiologic agent of cholera, the severe diarrhoeal disease attributable to the potent cholera toxin (CT) encoded by a prophage lysogenizing into the genome of the bacterium (Guerrant et al., 2003). Of the more than 200 serogroups identified, based on the variations of “O” antigenic lipopolysaccharide (LPS), *V. cholerae* serogroups O1 and O139 which possess CT can cause epidemic cholera. Historically *V. cholera*e serogroup O1 strains have two major biotypes, classical and El Tor, which differ in major phenotypic and genetic traits (Safa et al., 2010). *V. cholerae* O1 strains have three serotypes- Ogawa, Inaba, and Hikojima (Stroeher et al., 1992). *V. cholerae* O1 classical biotype caused the first six out of seven cholera pandemics before being replaced with the El Tor biotype which has been responsible for the ongoing seventh pandemic since 1961. The El Tor biotype strains have undergone genetic changes such as a new hybrid El Tor carrying the classical biotype CT (Nair et al., 2006).

The scientific literature suggests that *V. cholerae* transmits fecal-orally through contaminated water (Snow, 1855) following a “slow” human-to-aquatic environment-to-human pathway (Morris, 2011). But recent reports of hyper infectious strains suggest a potential alternative. Hyper infectivity is the term for a secondary physiologically altered state where isolates from one infected individual are more infectious for the immediate next individual. The literature suggests these strains might cause more severe disease and be capable of spreading rapidly following a faster “human-to-human” transmission route through fecal-oral contamination (Merrell et al., 2002; Morris, 2011). Therefore, cholera patients and the environmental reservoir can be considered as a potential source of outbreak but their respective relations to cholera transmission have been heavily disputed. Furthermore, in spite of the substantial work done in this field, the exact source and mode of transmission for amplification of the disease to reach an epidemic status is still not completely elucidated.

Dhaka, the capital city of Bangladesh, is one of the world's fastest growing megacities with an estimated population of more than 15 millions (World Bank, 2014). The city has undergone rapid urbanization over the past decades and population growth in the slums has increased exponentially (Das, 2000). According to the Dhaka Water Supply and Sewerage Authority (DWASA), many of the city's poorest households lack access to legal connections to DWASA water because they are squatting on illegal settlements. Therefore, water for drinking and daily use is often in short supply in these households, although measures have been undertaken recently to improve this situation (Wateraid, 2016). These conditions of water scarcity are ideal for the spread of cholera.

Cholera is endemic in Dhaka and the households of a cholera patient present an opportunity to investigate the transmission of cholera (Patel et al., 2012). Previous studies in Bangladesh have demonstrated that household contacts of cholera patients are at >100 times higher risk of cholera infections during the 1 week period after the index patient seeks hospital care (George et al., 2016). Multiple infections within the same household are common (Mosley et al., 1968; Weil et al., 2009), with approximately 17–29% of the contacts of cholera patients developing a *V. cholerae* infection (Kendall et al., 2010; Weil et al., 2014). However, our knowledge about the environmental transmission routes for the bacterium within the households of cholera patients is limited. We are aware of only one study to date that has analyzed water samples from households of cholera patients for isolation of *V. cholerae* (Spira et al., 1980). In the present study, we targeted the hospitalized cholera patients and investigated the transmission routes for cholera bacterium in the households of cholera patients in Dhaka city. This work will inform the development of future interventions to protect this high risk population.

## Materials and methods

### Study design: methods

Informed consent was obtained from all study participants (household contacts and index cholera cases), this included adult participant (≥18 years of age) signing an informed consent and/or parental consent form and children between the ages of 12 and 17 years old signing an assent form. If a study participant could not read, the consent form was read to the participant, then the participant was asked to document their consent with an x in the presence of a witness. All study procedures were approved by the research Ethical Review Committee of the International Centre for Diarrhoeal Disease Research, Bangladesh (icddr,b) and IRB of the Johns Hopkins Bloomberg School of Public Health.

The CHoBI7 randomized controlled trial (RCT) in Dhaka, Bangladesh was conducted from June 2013 to November 2014. A description of the intervention provided is described elsewhere (George et al., 2016). Suspected cholera cases, defined as patients presenting at the icddr,b Dhaka hospital with acute watery diarrhea (3 or more loose stools over a 24 h period) and moderate to severe dehydration using the WHO definition, were screened for the presence of *Vibrio cholerae* in their stool using the Crystal VC Rapid Dipstick test (Span Diagnostics, Surat, India) (WHO, 2005; George et al., 2014). All positive findings by dipstick were confirmed by bacterial culture. All suspected cholera cases admitted to icddr,b Dhaka hospital residing within a police station (thana) of Dhaka city were screened for eligibility for the CHoBI7 trial. Cholera cases were defined as suspected cholera cases with a stool bacterial culture result positive for *V. cholerae*. Cholera cases were excluded from the study if they had a household contact already enrolled (currently or previously), or if they had received cholera vaccine, to avoid confounding from an ongoing vaccine trial. Household contacts were defined as individuals sharing the same cooking pot as the index cholera case for the past 3 days. To be eligible for the study household contacts had to plan to reside in the household of the index case for the next week, and had not received cholera vaccine. Eligible household contacts present in the hospital at the time of case enrollment were invited to participate, and a household visit was made to recruit household contacts within 36 h of case enrollment. A cluster was defined as the index cholera case and their corresponding household contacts.

Case households were visited at Days 1, 3, 5, 7, and 9 (Visits 1–5) after the presentation of the cholera case at icddr,b Dhaka Hospital for clinical and environmental surveillance. For clinical surveillance, household contacts were asked if they had diarrhea (3 or more loose stools over a 24 h period) or vomiting in the past 48 h, and a rectal swab sample was collected from willing household contacts at each household visit to test for the presence of *V. cholerae* in stool by bacterial culture. For environmental surveillance, a water sample was collected from the household's water source and stored drinking water in the home at each household visit to test for the presence of *V. cholerae* by bacterial culture. Thirty three households from the main CHoBI7 trial were randomly selected for this sub-study. For water sources and household members with multiple *V. cholerae* O1 strains, only one strain was randomly selected. In addition, four *V. cholerae* strains from water bodies of circular river systems of Dhaka city isolated in 2013 (*n* = 2) and 2014 (*n* = 2) that were separate from the main randomized controlled trial were included in the analysis as representative environmental strains.

### Sample collection and processing

Stool samples from the cholera patients (index patient) were collected at icddr,b Dhaka hospital. Rectal swab samples were collected in Cary-Blair media and water samples were collected in 500 mL bottles and transported to the icddr,b Dhaka laboratory for analysis. Water samples were filtered through 0.22 μm filter papers and the membrane filters were then enriched in alkaline peptone water (APW) (pH 8.4) at 37°C for 4–6 h, and then cultured on selective media, as described previously (Alam et al., 2007). Stool and rectal swabs were also subjected to APW enrichment and subsequent culture following same procedure.

### Isolation and identification of *V. cholerae*

After culturing on selective media, isolation and identification of typical *V. cholerae* colonies were performed according to standard methods (Huq et al., 2012). The serogroups of the *V. cholerae* strains were determined serologically by a slide agglutination test using specific polyvalent antisera for *V. cholerae* O1 and O139. The serotypes of these strains were confirmed using serotype-specific monovalent Inaba and Ogawa antisera (Alam et al., 2007). Biotyping was performed using a number of phenotypic tests: chicken erythrocyte agglutination (chicken cell agglutination; CCA), sensitivity to polymyxin B, and Mukerjee CL phage IV and Mukerjee ET phage V tests (Kaper et al., 1995).

### PCR assays

All strains that were preliminarily identified as *V. cholerae* were reconfirmed using a *V. cholerae* species-specific *ompW* PCR. Multiplex PCR assays were performed to identify genes encoding O1- (*rfbO1*) and O139- (*rfbO139*) specific O biosynthetic genes, as well as the virulence gene *ctxA* (Hoshino et al., 1998). To complement the biotype characterization by phenotypic traits, PCR assays were performed using previously described procedures that were targeted to detect the *tcpA* allele (CL and ET) (Rivera et al., 2001), the type of the *rstR* gene and presence of *rstC* gene encoding the phage transcriptional regulator (Kimsey and Waldor, 1998), and the *rtxC* gene of RTX (repeat in toxin) (Chow et al., 2001). Double mismatch amplification mutation assay (DMAMA)-PCR was used to distinguish between the CL (*ctxB* genotype 1), ET (*ctxB* genotype 3) and Haitian types (*ctxB* genotype 7) of ctxB alleles by focusing on nucleotide positions 58 and 203 of the *ctxB* gene. DMAMA-PCR was performed in this study to detect the *ctxB* genotype using the primers and conditions described elsewhere (Naha et al., 2012).

### Pulsed-field gel electrophoresis (PFGE)

The whole agarose-embedded genomic DNA for *V. cholerae* was prepared. PFGE was carried out with a contour-clamped homogeneous electrical field (CHEF-DR II) apparatus (Bio-Rad), according to procedures described elsewhere (Cooper et al., 2006). Genomic DNAs of the test strains were digested by the *Not*I restriction enzyme (Gibco-BRL), and *Salmonella enterica* serovar Braenderup was digested by *Xba*I, with the fragments being used as molecular size markers. The restriction fragments were separated in 1% pulsed-field-certified agarose in 0.5X TBE (Tris/borate-EDTA) buffer. In the post electrophoresis gel treatment step, the gel was stained and de-stained. The DNA was visualized using a UV trans illuminator, and images were digitized by a 1D gel documentation system (Bio-Rad). The fingerprint pattern in the gel was analyzed using the Bionumeric software (Version 3.1). Dendrogram was constructed on the basis of banding similarity and dissimilarity using the Dice similarity coefficient and unweighted-pair group method (UPGMA) as recommended by the manufacturer.

## Results

### Analysis of bacterial strains from subset households

Of total 420 rectal swab samples analyzed from 84 household contacts and 330 water samples (165 source water and 165 household stored drinking water samples) collected from 33 households, *V. cholerae* was isolated from 20%(17/84) of household contacts, 18%(6/33) of stored drinking water, and 27%(9/33) of source water samples. Out of the 33 cholera patient households, we found 15%(5/33) of households with *V. cholerae* isolated from both household contacts and water samples, 21%(7/33) of households with *V. cholerae* only isolated from household contacts, 18%(6/33) of households with *V. cholerae* only isolated from water, and 45%(15/33) of households with no household contact or water samples with detectable *V. cholerae* (Table 1). Six percent(2/33) households had detectable *V. cholerae* in household stored water, 12%(4/33) households had detectable *V. cholerae* in source water, and 15%(5/33) households had detectable *V. cholerae* in both source water and household stored water.

**Table 1:** **Molecular characteristics of ***Vibrio cholerae*** O1 isolated from Cholera patients, their family members and drinking water sources**.

**Cluster no**.	**Police station**	**Year of isolation**	**Source**	**Serotype**	***rfb*O1**	***ctxA***	***ctxB* genotype**	***rstR* type**	***tcpA* type**	***rstC***	**Biotype**
C4	Khilgaon	2013	IP	Ogawa	+	+	B1	ET	ET	+	Al-ET
C5	Bangshal	2013	IP	Ogawa	+	+	B1	ET	ET	+	Al-ET
		2013	SW	Ogawa	+	+	B1	ET	ET	+	Al-ET
		2013	HC	Ogawa	+	+	B1	ET	ET	+	Al-ET
		2013	HC	Ogawa	+	+	B1	ET	ET	+	Al-ET
C7	Vatara	2013	IP	Ogawa	+	+	B1	ET	ET	+	Al-ET
		2013	SW	Ogawa	+	+	B1	ET	ET	+	Al-ET
		2013	HC	Ogawa	+	+	B1	ET	ET	+	Al-ET
		2013	HC	Ogawa	+	+	B1	ET	ET	+	Al-ET
C9	Badda	2013	IP	Ogawa	+	+	B1	ET	ET	+	Al-ET
C11	Tejgaon	2013	IP	Ogawa	+	+	B1	ET	ET	+	Al-ET
		2013	SW	Ogawa	+	+	B1	ET	ET	+	Al-ET
		2013	HW	Ogawa	+	+	B1	ET	ET	+	Al-ET
C16	Mohammadpur	2013	IP	Ogawa	+	+	B1	ET	ET	+	Al-ET
		2013	SW	Ogawa	+	+	B1	ET	ET	+	Al-ET
		2013	HW	Ogawa	+	+	B1	ET	ET	+	Al-ET
		2013	HC	Ogawa	+	+	B1	ET	ET	+	Al-ET
		2013	HC	Ogawa	+	+	B1	ET	ET	+	Al-ET
C18	Mirpur	2013	IP	Ogawa	+	+	B1	ET	ET	+	Al-ET
		2013	SW	Ogawa	+	+	B1	ET	ET	+	Al-ET
		2013	HC	Ogawa	+	+	B1	ET	ET	+	Al-ET
C19	Banani	2013	IP	Ogawa	+	+	B1	ET	ET	+	Al-ET
		2013	HC	Ogawa	+	+	B1	ET	ET	+	Al-ET
C20	Badda	2013	IP	Ogawa	+	+	B1	ET	ET	+	Al-ET
		2013	HC	Ogawa	+	+	B1	ET	ET	+	Al-ET
C21	Mohammadpur	2013	IP	Ogawa	+	+	B1	ET	ET	+	Al-ET
		2013	HC	Ogawa	+	+	B1	ET	ET	+	Al-ET
C23	Pallabi	2013	IP	Ogawa	+	+	B1	ET	ET	+	Al-ET
C24	Adabor	2013	IP	Ogawa	+	+	B1	ET	ET	+	Al-ET
		2013	HC	Ogawa	+	+	B1	ET	ET	+	Al-ET
C25	Mirpur	2013	IP	Ogawa	+	+	B1	ET	ET	+	Al-ET
		2013	SW	Ogawa	+	+	B1	ET	ET	+	Al-ET
		2013	HW	Ogawa	+	+	B1	ET	ET	+	Al-ET
C26	Hazaribag	2013	IP	Ogawa	+	+	B1	ET	ET	+	Al-ET
C30	Sher-E-Bangla Nagar	2013	IP	Ogawa	+	+	B1	ET	ET	+	Al-ET
C31	Mohammadpur	2013	IP	Ogawa	+	+	B1	ET	ET	+	Al-ET
		2013	HC	Ogawa	+	+	B1	ET	ET	+	Al-ET
C33	Cantonment	2013	IP	Ogawa	+	+	B1	ET	ET	+	Al-ET
C35	Gulshan	2013	IP	Ogawa	+	+	B1	ET	ET	+	Al-ET
C39	Mirpur	2013	IP	Ogawa	+	+	B1	ET	ET	+	Al-ET
		2013	HW	Ogawa	+	+	B1	ET	ET	+	Al-ET
		2013	HC	Ogawa	+	+	B1	ET	ET	+	Al-ET
		2013	HC	Ogawa	+	+	B1	ET	ET	+	Al-ET
C41	Tejgaon	2013	IP	Ogawa	+	+	B1	ET	ET	+	Al-ET
		2013	HC	Ogawa	+	+	B1	ET	ET	+	Al-ET
		2013	HC	Ogawa	+	+	B1	ET	ET	+	Al-ET
C42	Sher-E-Bangla Nagar	2013	IP	Ogawa	+	+	B1	ET	ET	+	Al-ET
		2013	SW	Ogawa	+	+	B1	ET	ET	+	Al-ET
C47	Kafrul	2013	IP	Ogawa	+	+	B1	ET	ET	+	Al-ET
		2013	HC	Ogawa	+	+	B1	ET	ET	+	Al-ET
C48	Rampura	2013	IP	Ogawa	+	+	B1	ET	ET	+	Al-ET
C50	Shabujbag	2013	IP	Ogawa	+	+	B1	ET	ET	+	Al-ET
C52	Malibag	2013	IP	Ogawa	+	+	B1	ET	ET	+	Al-ET
		2013	HW	Ogawa	+	+	B1	ET	ET	+	Al-ET
C53	Vashantek	2013	IP	Ogawa	+	+	B1	ET	ET	+	Al-ET
C75	Mugda	2014	IP	Ogawa	+	+	B1	ET	ET	+	Al-ET
C80	Dhanmondi	2014	IP	Ogawa	+	+	B1	ET	ET	+	Al-ET
C89	Motijheel	2014	IP	Ogawa	+	+	B1	ET	ET	+	Al-ET
C92	Lalbagh	2014	IP	Ogawa	+	+	B1	ET	ET	+	Al-ET
C101	Rupnagar	2014	IP	Ogawa	+	+	B1	ET	ET	+	Al-ET
		2014	SW	Ogawa	+	+	B1	ET	ET	+	Al-ET
C120	Kalabagan	2014	IP	Ogawa	+	+	B1	ET	ET	+	Al-ET
C153	Lalbagh	2014	IP	Ogawa	+	+	B1	ET	ET	+	Al-ET
		2014	SW	Ogawa	+	+	B1	ET	ET	+	Al-ET
		2014	HW	Ogawa	+	+	B1	ET	ET	+	Al-ET

*IP, Index Patient (First Cholera patient from a family); HC, Household Contact (Family member of Cholera patient); SW, Drinking Water (Source); HW, Drinking Water (Household); ET, El-Tor; rfbO1, O1 antigen coding gene; ctxA, Cholera Toxin A subunit; ctxB, Cholera Toxin B subunit; rstR, Repeat Sequence Transcription Repressor gene; tcpA, Toxin Co-regulated Pilus subunit A; rstC, Repeat Sequence Transcription Anti-repressor gene*.

**Figure 1:**
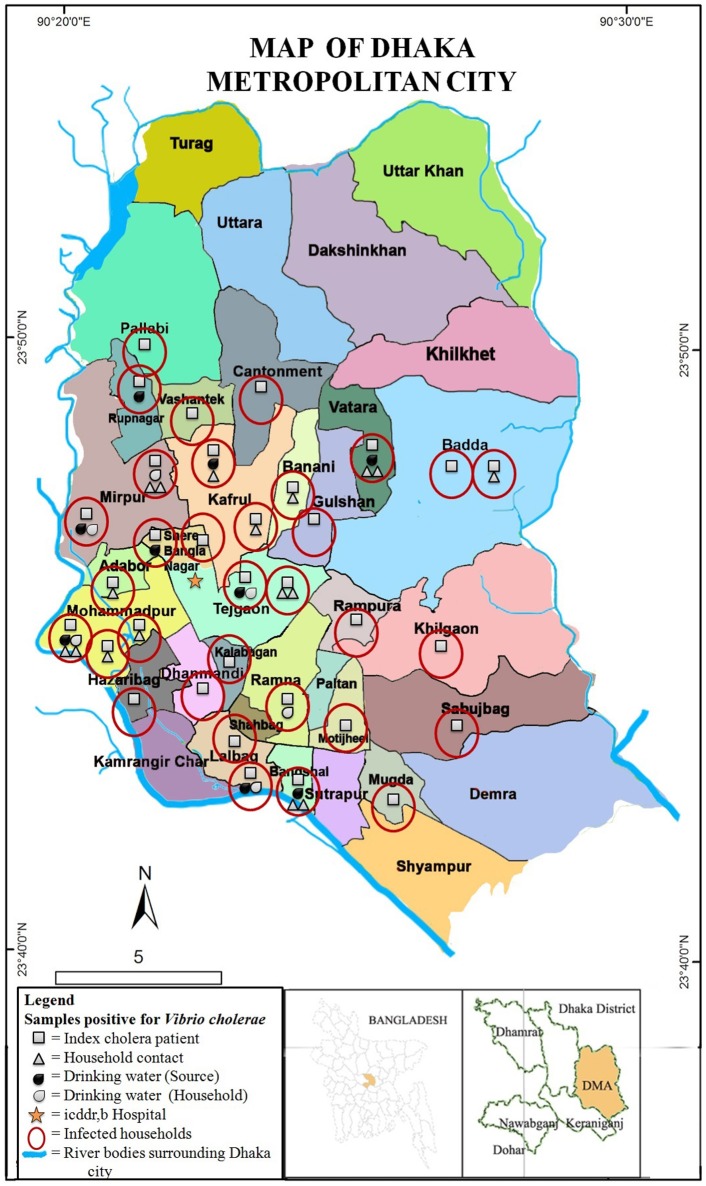
**Map showing distribution of households with ***Vibrio cholerae*** O1 strains isolated from hospitalized cholera patients, their household members, and drinking water sources in different police stations of Dhaka city**. Drinking water samples from both source and household storage were included. All samples were collected from 33 households of cholera patients, distributed across 25 police stations (thana) of the city.

A map was prepared showing the distribution of the 33 households of cholera patients across Dhaka city (Figure 1). The map includes information on whether household contacts, water source, and stored water samples had detectable *V. cholerae*. The map showed that households of cholera patients were distributed widely across the 25 police stations (thana), and covered majority of the Dhaka metropolitan areas (Figure 1). Households with multiple *V. cholerae* infected household members and water samples were clustered in the south-western part of the city, with the majority residing in slum areas with residents of low socioeconomic status (Personal Communication: Dr. Munirul Alam). There was also a higher proportion of cholera patients and infected household members in the south-western part of the city close to the heavily polluted circular rivers and water bodies.

One exception, however, was a household with multiple infected household contacts located in the north-eastern police station of Vatara. This household was located in an area next to a lake which was adjacent to a relatively affluent area. The households located in the eastern parts of the city, mostly had only infected index cholera patients. The majority of cholera patient households had relatively low socioeconomic status with 15% owning a refrigerator, and 61% owning a television. Fifty two percent of households reported Dhaka Water Supply and Sewerage Authority (DWASA) water as their primary drinking water source. However, based on the depth of the ground water aquifer in Dhaka and maps of the distribution of DWASA water we suspect all households were actually utilizing DWASA water, and were confused about the source of their drinking water.

### Phenotypic and molecular characterization of *V. cholerae*

*V. cholerae* isolates were serologically confirmed to be *V. cholerae* O1, and all possessed “O” serogoup-specific gene *rfbO1*, further confirming the serological results (Table 1). All *V. cholerae* strains belonged to serotype Ogawa; and all possessed the cholera toxin gene, *ctxA* confirming they were toxigenic. In addition, all *V. cholerae* O1 strains possessed ET biotype-specific toxin co-regulated pilus (*tcpA*^*ET*^), phage transcription regulation gene (*rstR*^*ET*^), phage transcription anti-repressor gene (*rstC*), and a repeat in toxin (*rtxC*), confirming El Tor biotype traits.

The *ctxB* genotype of *V. cholerae* O1 was determined by mismatch amplification mutation assay (MAMA)-PCR, and double mismatch amplification mutation assay (DMAMA)-PCR, which showed that all *V. cholerae* strains possessed *ctxB* genotype 1, which is the classical type CT, confirming that the bacterium was El Tor but possessed classical biotype CT.

### PFGE of *Not*I-digested genomic DNA

**Figure 2:**
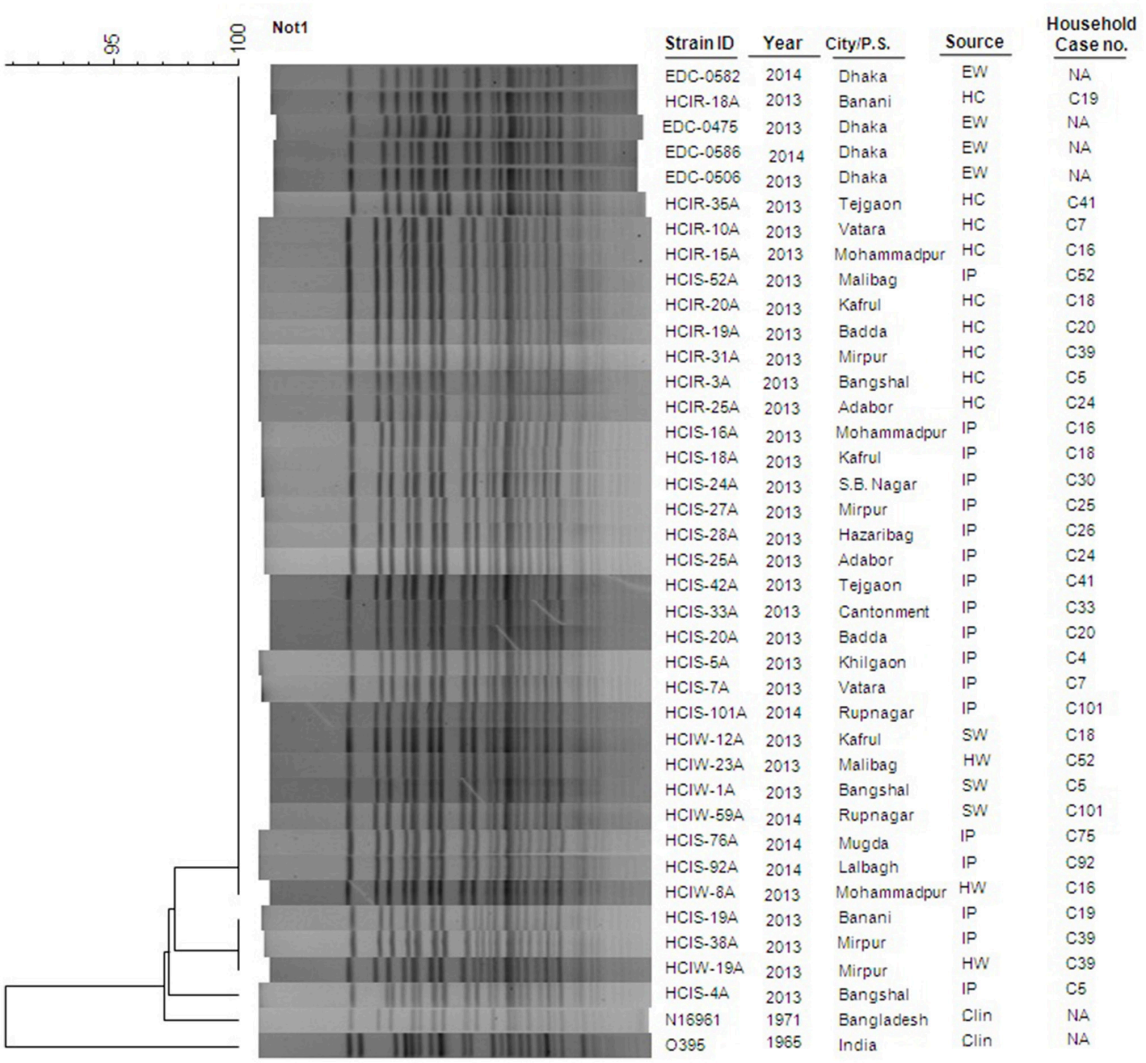
**Genetic Characterization using PGFE of water and clinical ***V. cholerae*** O1 strains collected in the households of cholera patients**. Thirty seven *V. cholerae* O1 strains were isolated in Dhaka city from hospitalized cholera patients (*n* = 18), their household contacts (*n* = 9), drinking water sources (*n* = 6) and environmental water (*n* = 4) from waterbodies circulating around the city. The dendrogram was constructed by Dice similarity coefficient and mainstream hierarchical clustering (UPGMA) using PFGE images of *Not*1-digested genomic DNA. The scale bar at the top left indicates the similarity coefficient (range: 90–100%). IP, Index Patient; HC, Household contact; EW, Environmental Water; SW, Source Water; HW, House-hold Water; Clin, Clinical; NA, Not Applicable.

Of the 33 *V. cholerae* O1 strains tested by PFGE, 88% (29/33) of strains had identical banding pattern (Figure 2) and all belonged to the same clonal complex. For the four strains with different PFGE patterns, all exhibited 95% similarity when cluster analysis was done by dendrogram (Figure 2). Another four *V. cholerae* O1 strains isolated in 2013 (*n* = 2) and 2014 (*n* = 2) from water of circular river systems of Dhaka city had identical PFGE pattern, and shared a cluster with the majority of strains which proved to be clonal. The N16961 (*V. cholerae* prototype El Tor) and O395 (*V. cholerae* prototype classical) reference strains showed significant difference with the altered El Tor strains analyzed in the present study.

## Discussion

The data presented on the phenotypic and molecular characteristics confirmed all *V. cholerae* strains to be serogroup O1, serotype Ogawa, and biotype El Tor (ET) possessing classical biotype cholera toxin (CT). Furthermore, all strains possessed *rstC* and had *rstR* and *tcpA* that were of ET biotype confirming that all were altered ET. The *ctxB* typing results confirmed the *V. cholerae* O1 strains to possess *ctxB*1 in Dhaka city. This is consistent with results of a recent study demonstrating a genetic shift in *ctxB*, from *ctxB*7 to *ctxB*1 (Rashid et al., 2015), but not *ctxB*7 as reported previously from Dhaka city (Rashed et al., 2012). Furthermore, our molecular typing data obtained from pulsed-field gel electrophoresis (PFGE) suggest the transmission of a single infectious *V. cholerae* clone selectively circulating through drinking water following a fecal-oral mode of transmission in Dhaka city.

Consistent with the literature we found that 20% of household contacts of cholera patients were positive for *V. cholerae* during our 1 week surveillance period (Spira et al., 1980; Weil et al., 2009). These findings further demonstrate the high risk of cholera among this population. We suspect this high risk is mostly due to the rapid spread of hyperinfectious strains within study households. Furthermore, drinking water samples collected from both DWASA and household stored water had detectable toxigenic *V. cholerae* O1. The highest incidence was in source water (27%) compared to stored drinking water (18%). Therefore our data shows that drinking water in households of cholera patients is contaminated and can serve as source of toxigenic *V. cholerae* O1 in Dhaka city. In addition, the presence of family members with asymptomatic cholera infections can put other household members at a high risk of cholera disease.

We observed in our map that cholera patients were distributed across Dhaka with the majority of cholera patient households being of low socioeconomic status. The majority of cholera patients were found in the western part of the city in Mirpur, Mohammadpur, and adjacent areas which have had rapid urbanization in the past few years. These findings suggest that households of low socioeconomic status and those that do not have access to safe drinking water are at the highest risk of cholera. This is consistent with Luby et al. (2010) which found overcrowded living conditions with rapid population growth to be highly susceptible to infectious diseases. Household contacts of patients coming from affluent parts of Dhaka did not develop cholera infections. This is presumable because the index patient drank contaminated water or food from outside their home. In the present study, many households (49%) reported dugwells as their primary drinking water source. But, it is very highly unlikely as the underground water level continues to decline in Dhaka city, and far beyond the dugwell range. We suspect that the dugwells in slum households preserve DWASA water that they access through illegal connections (Personal Communication: Dr. Munirul Alam).

Despite significant achievements in the millennium development goals, and public health being a key priority for the government, recurrent cholera continues to cause substantial morbidity and mortality in Bangladesh. *V. cholerae*, the causative agent of cholera, has been well established as the native flora of aquatic environments and drinking water has been found as a risk factor for cholera (Alam et al., 2006). Boucher et al. (2015) proposes that large-scale human activities in densely-populated areas and being situated near the low-lying Ganges delta are important factors in the evolution of *V. cholerae* in countries such as Bangladesh. This theory is plausible for Dhaka as it is surrounded by waterbodies and has the highest population growth rate in the world (World Bank, 2014). Cholera is endemic in Bangladesh and outbreaks are generally associated with a single yearly peak in the coastal villages and bimodal peaks in urban Dhaka presumably due to its high population density and heavily contaminated water bodies and flood embankments (Alam et al., 2011). Ultimately all these factors contribute to cholera being present in Dhaka city throughout the year. Trapped in a continuous cycle of accelerated transmission, the organism likely does not have the opportunity to revert back to non-toxigenic or in the viable but non-culturable (VBNC) state (Colwell, 1996) and presumably remains locked-up in the continuous fecal-oral mode of transmission in the urban ecosystem.

## Conclusion

Cholera is a serious public health threat globally, especially in highly populated urban setting such as Dhaka where the disease is endemic and rapid unplanned urbanization and poor water and sanitation conditions are favorable for clonal transmission of *V. cholerae*. Under the changing climate and the growing urban slum population in Dhaka city, safe water and hygiene interventions are urgently needed to prevent millions from cholera and other infectious diseases. Thus, delivering evidence based WASH interventions to promote hand-washing with soap and water treatment, and the provision of cholera vaccination is essential for combating cholera globally.

## Author contributions

MA and CG are the Principal Investigators of the project and contributed to the design of the study, manuscript revision and final approval of version to be published. SM is the functional Principal Investigator of the project and contributed to the design of the study, manuscript review and critical revision. RR and MR designed and implemented the study. RR performed the study in the laboratory and wrote the first draft of the manuscript. DS, RS contributed to the study designing and manuscript writing and revising it critically for important intellectual content. SR contributed to revising the manuscript critically for important intellectual content. ZR, MTM, MM, FJ, and SI performed the study in the laboratory and reviewed the manuscript. KS and MS oversaw collection of data in the hospital/field and reviewed manuscript. TP was involved in data collection and contributed in manuscript writing. MB was involved in database construction and statistical analysis and reviewed the manuscript. All authors read and approved the final manuscript. The authors have agreed to be accountable for all aspects of the work in ensuring that questions related to the accuracy or integrity of any part of the work were appropriately investigated and resolved.

## Funding

All strains were collected during the study entitled as “Cholera Hospital Based Intervention for 7 Days (CHoBI7)” conducted in International Centre for Diarrheal Disease Research, Bangladesh (icddr,b) in Dhaka, Bangladesh; funded by National Institute of Health (NIAID-R01AI039129/NIAID-1K01AI110526). The icddr,b is also thankful to the governments of Australia, Bangladesh, Canada, Sweden and the UK for providing core/unrestricted support.

### Conflict of interest statement

The authors declare that the research was conducted in the absence of any commercial or financial relationships that could be construed as a potential conflict of interest.
